# A Comparison Between Ecological Momentary Assessment and the Adapted-Quick Drinking Screen: Alcohol Mixed With Energy Drinks

**DOI:** 10.1093/alcalc/agab086

**Published:** 2022-01-17

**Authors:** Sean J Johnson, Joris C Verster, Chris Alford

**Affiliations:** Centre for Trials Research, Cardiff University, Cardiff CF14 4YS, UK; Psychological Sciences Research Group, University of the West of England, Bristol BS16 1QY, UK; Division of Pharmacology, Utrecht Institute for Pharmaceutical Sciences (UIPS), Utrecht University, Utrecht 3584CG, The Netherlands; Centre for Human Psychopharmacology, Swinburne University, Melbourne, VIC 3122, Australia; Psychological Sciences Research Group, University of the West of England, Bristol BS16 1QY, UK; Centre for Human Psychopharmacology, Swinburne University, Melbourne, VIC 3122, Australia

## Abstract

**Aims:**

To compare alcohol consumption and risk-taking behaviours on alcohol mixed with energy drink (AMED) and alcohol-only (AO) drinking occasions collected via ecological momentary assessment (EMA) versus retrospective survey methods (adapted-Quick Drinking Screen: a-QDS).

**Methods:**

Completing participants were 52 university students who reported AMED consumption during the 30-day data collection period. Alcohol consumption and risk-taking behaviours were captured for recreational AMED and AO consumption occasions using a smartphone-based app across 30 days. Data were aggregated for comparison with the a-QDS conducted at the end of data collection.

**Results:**

Irrespective of data collection method, alcohol was consumed more frequently and at higher quantities on the heaviest drinking occasions when consumed alone compared with when it was mixed with energy drinks. Consistent with this finding, more risk-taking behaviours were experienced on AO occasions compared with AMED occasions. Compared with the a-QDS, the quantity of alcohol consumed on the average and heaviest drinking occasion was significantly higher when reported via EMA. This was consistent across both AO and AMED drinking occasions.

**Conclusion:**

EMA provides a more valid measure of consumption quantity compared with retrospective recall, which was susceptible to under-reporting, although this was not differentially affected across consumption occasions. In line with previous research, this study demonstrated that mixing alcohol with energy drinks does not increase alcohol consumption or risk-taking behaviours.

## Teaser

**Short Summary:** Retrospective recall of alcohol consumption quantity is susceptible to under-reporting in comparison with ecological momentary assessment, this is not differentially affected across consumption occasions. Mixing alcohol with energy drinks does not increase alcohol consumption or risk-taking behaviours compared with drinking alcohol alone.

## INTRODUCTION

Since their introduction to the beverage market in the 1980s, energy drinks have become increasingly popular, particularly among young adults and adolescents. Energy drinks are non-alcoholic beverages that contain caffeine and other ingredients, typically B-vitamins and taurine, with motives for consumption including to keep consumers awake, liking the taste, to provide energy and to increase alertness and concentration (Johnson *et al*., 2016a).

As the popularity of energy drinks has grown, so has the consumption of alcohol mixed with energy drinks (AMED). Although it is difficult to draw an accurate picture of AMED prevalence due to the different methodologies used (i.e. different questionnaires, scale types and intervals), a recent review of studies focusing mainly on university students (aged 19–25 years) concluded past month AMED consumption to be between 14.7 and 26.0% of the student population (Vida and Racz, 2015). However, higher consumption levels have been reported in other countries including the UK, with 39% of students reporting past month AMED consumption (Johnson *et al*., 2016b).

Over the past decade a growing body of research has investigated this popular consumption practice. In particular, concerns have been raised regarding the potential pharmacological interaction of caffeine with alcohol. It has been suggested that this interaction may result in reduced perception of alcohol intoxication, or masking effect, resulting in greater alcohol consumption and more negative alcohol-related consequences (Arria and O’Brien, 2011; Peacock *et al*., 2014). Survey research has consistently demonstrated that AMED consumers are more likely to drink higher volumes of alcohol and engage in risky behaviours compared with those who consume alcohol-only (AO; O’Brien *et al*., 2008; Woolsey *et al*., 2010; Brache and Stockwell, 2011; de Haan *et al*., 2012; Eckschmidt *et al*., 2013; Lubman *et al*., 2013; Trapp *et al*., 2013; Vida and Racz, 2015; Woolsey *et al*., 2015a, 2015b; Johnson *et al*., 2016b). However, laboratory studies investigating the interaction effects of caffeine within energy drinks and alcohol have failed to support the notion of a masking effect. Indeed a recent systematic review and meta-analysis concluded that consuming alcohol with caffeinated beverages does not impair judgement of subjective intoxication (Benson *et al*., 2014). These contrasting findings led some researchers (Verster *et al*., 2012) to consider the possibility that AMED consumption is mediated by a third ‘trait’ factor, i.e. individuals who are drawn to consume this beverage mix may also be heavier alcohol consumers and higher risk-takers, thus explaining the perceived increase in these behaviours when examined using between-subjects designs. Research has consistently found that AMED consumers differ in many personality and behavioural aspects to those that do not consume AMED, including increased levels of sensation seeking, smoking, drug use and unsafe sex (Verster *et al*., 2018).

In order to identify whether AMED contributes independently to increased alcohol consumption and risk-taking behaviours over and above personality traits, some researchers have employed within-subjects designs comparing AMED and AO drinking occasions in AMED consumer cohorts. Meta-analysis combining the results of these studies showed that mixing alcohol with energy drinks did not significantly increase overall alcohol consumption or increase the number of experienced negative alcohol-related consequences (Verster *et al*., 2018). Thus, whilst AMED consumers usually drink more alcohol than AO consumers, this is irrespective of whether energy drinks are consumed with alcohol ornot.

Despite consistent within-subject findings refuting the link between AMED use and alcohol outcomes, the majority of the available evidence has been based on cross-sectional designs asking participants to report on their typical or past month/year AMED and AO use. Although cross-sectional designs are advantageous in being quick to conduct, convenient for participants and cost-effective, retrospective methods of collecting data do have limitations. The ability to accurately recall the number and type of drinks consumed, as well as the number of negative alcohol-related consequences experienced, is likely to be affected by the amount of time passed as well as alcohol-related amnesic effects (Verster *et al*., 2003; Platt *et al*., 2016). Some researchers have suggested that this recall bias could differentially affect memory of AMED occasions over AO occasions (Linden-Carmichael *et al*., 2018). A potential mechanism for this effect is that as AMED consumers tend to drink AO on the majority of drinking occasions, there is more opportunity to recall AO-related harm (Scholey *et al*., 2018). However, no research has systematically exploredthis.

To address the potential inaccuracy associated with retrospective methods, some researchers have adopted daily diary designs. This involves participants reporting on their alcohol consumption and outcomes close to the time they occur. This typically occurs the day after an alcohol consumption occasion. To date, two studies have used this method to investigate AMED consumption. In order to compare drinking days where individuals did versus did not consume energy drinks, Patrick and Maggs (2014) conducted 14-day bursts of daily surveys in four consecutive college semesters. They found that adding energy drink use to a day with alcohol use was associated with an increase in the number of alcoholic drinks, more time spent drinking, higher estimated blood alcohol content (eBAC), a greater likelihood of subjective intoxication and an increased number of negative alcohol-related consequences. However, after controlling for eBAC energy drink use no longer predicted subjective intoxication. A shortcoming of this study was that rather than directly assessing the simultaneous consumption of alcohol with energy drinks, it examined the extent to which drinking an energy drink at any time during a day increased the odds of experiencing certain alcohol-related outcomes. Thus, a drinking occasion could be defined as AMED even when the energy drink was consumed outside of the half-life of the proposed interactive substance caffeine of around 5 h (Nehlig, 2018). For example, consuming an energy drink during the morning to increase alertness at work and consuming alcohol in the evening at a night-time entertainment venue would be classified as an AMED drinking occasion.

More recently, Linden-Carmichael and Lau-Barraco (2017) used a 14-day diary study of 18–25 years old heavy drinking college students who reported past week consumption of caffeinated alcoholic beverages (CAB—including AMED as well as other mixers such as diet and regular sodas). On each day participants reported on their drinking behaviour, including the type and amount of alcohol consumed and harms experienced. Using multilevel modelling they found that participants consumed significantly more alcohol on occasions in which they consumed alcohol with caffeine (regardless of whether the mixer was an energy drink) in comparison with AO drinking occasions. In addition, after controlling for the amount of alcohol consumed, AMED but not cola caffeinated mixers, were associated with more alcohol-related harms than AO drinking occasions. Although this study is an important contribution to progressing AMED research, concerns have been raised regarding the data analysis and reporting (Scholey *et al*., 2018). In summary, the study examined only a small number of AMED drinking occasions (40 AMED occasions or 2.64% of consumption days available to the 122 AMED consumers across the average 12.42 entries), consisted of mainly female participants (73.8%), and included all drinking occasions (i.e. a glass of wine with a meal) rather than recreational alcohol consumption, therefore diluting alcohol consumption on AO drinking occasions.

However, the main limitation is that this study did not conduct true within-subject analysis but compared CAB with non-CAB occasions within a cohort of CAB users. A more appropriate approach would have been to compare alcohol consumption and consequences among only those participants that experienced both CAB (and more specifically AMED) and AO within the 14-day period. This operationalization of consumption groups by Linden-Carmichael and Lau-Barraco (2017) make it difficult to determine the role of AMED in contributing to alcohol-related harms.

Although daily diary studies are advantageous in reducing recall bias, there is still the potential that recall the following day is clouded by alcohol-related amnesic effects (Verster *et al*., 2003), particularly at high levels of alcohol consumption. Daily diary studies are also subject to participant compliance issues, such as completing the questionnaire at a later date rather than when engaging in the behaviour. This was illustrated by Stone *et al*. (2003) who used a light sensor to detect when participants opened the diary and recorded their consumption. On average participants reported a 90% compliance rate, whereas the light sensor revealed compliance to be only 11%.

An alternative method more recently employed is to use ecological momentary assessment (EMA). This involves capturing ‘life as it is lived’ (Bolger *et al*., 2003) by recording individuals’ behaviour in their environments and over time (Trull and Ebner-Priemer, 2014). The increased use and versatility of smartphones has enabled the study of a variety of problematic behaviours, such as alcohol consumption (Kuntsche and Labhart, 2013; Clapp *et al*., 2017; Dulin *et al*., 2017; Merrill *et al*., 2017) and illegal drug use (Kennedy *et al*., 2013) in real-time. This approach has been shown to provide more nuanced information in comparison with retrospective measures. For example, Poulton *et al*. (2018) found an increase in the number of drinking days and higher alcohol intake via a smartphone-based app compared with the alcohol timeline followback (TLFB). However, to date no research has utilized this methodology to investigate the effects of mixing alcohol with energy drinks.

Given the limitations of previous daily diary studies and the need to assess the accuracy of retrospective survey methods, which underpin the majority of AMED research findings, the aim of the present study was to compare alcohol consumption and risk-taking behaviours on AMED and AO drinking occasions collected via EMA (smartphone-based app) versus retrospective survey assessment (adapted-Quick Drinking Screen, a-QDS).

## METHOD

### Participants and procedure

Following initial ethics approval (HAS/16/03/116) study participants were recruited via the University of the West of England (UWE) Student Union social media, on campus advertising and word of mouth. Students who were interested in taking part in the study were provided with an online link to complete the screening questionnaire. Exclusion criteria included being pregnant or breastfeeding, taking prescription medication (excluding contraceptive pill) and having any underlying health issues. Inclusion criteria included being a student at the University of the West of England (UWE), aged between 18 and 35 years old and having a mobile device with internet access. In addition, to be eligible participants had to report at least two AMED and two AO drinking occasions in the past 2 months. This was to ensure that sufficient data were collected to make meaningful within-subject comparisons between AMED and AO drinking occasions. Eligible participants were invited to attend baseline assessment at the University of the West of England Psychology laboratory. Here, participants were provided with further details on the purpose of the study and informed consent obtained. Participants were asked to complete a baseline questionnaire consisting of demographic information as well as details relating to medication, smoking, alcohol and drug use. Finally, participants were supported in downloading the smartphone-based app, if they did not already have this installed on their device, and were provided with training on how to submit alcohol consumption data. A recreational alcohol consumption occasion was defined as an event or activity with others where alcohol was consumed. This included, but was not limited to, drinking alcohol with friends or family at home, outdoors, at restaurants and at night-time entertainment venues e.g. pubs, bars and nightclubs. Alcohol consumption prior to going out (pre-drinking) was included within the recreational alcohol consumption occasion. The baseline assessment took ~30–45 min to complete.

Participants were then asked to utilize the smartphone-based app for a 2-week familiarization period. The purpose of this familiarization period was to pilot the smartphone-based app to see if it was feasible for both the researcher and participant to collect alcohol consumption data in this way. Based on previous research (Johnson *et al*., 2016b) this timeframe was deemed sufficient to capture two or more alcohol consumption occasions per participant. At the end of this 2-week familiarization period, participants were contacted via smartphone and any user-acceptance issues addressed. If willing, participants were then asked to continue using the smartphone-based app to record their alcohol consumption over a 30-day period.

**Figure 1:**
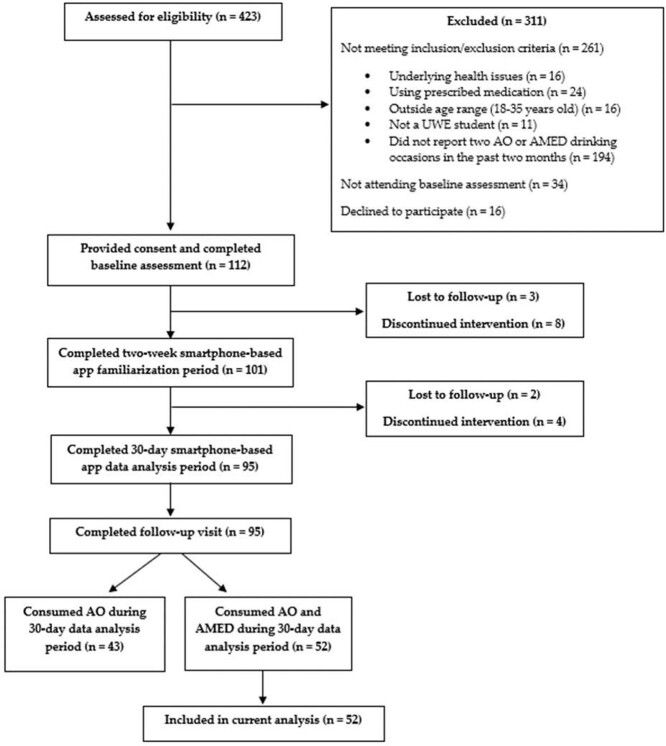
Consort flow diagram.

To conclude, the study participants were asked to attend the laboratory for a follow-up visit. Here, participants completed alcohol consumption questions (Sobell *et al*., 2003) as well as risk-taking behaviours (Peacock *et al*., 2012) with reference to the past 30-days. Participants were provided with feedback on their alcohol consumption and risk-taking levels and signposted to advice services if required. The follow-up visit took ~30–45 min to complete. Participants were reimbursed £50 for taking the time to participate in the study. Figure 1 shows the flow of participants through each stage of the study.

### Measures

#### Baseline assessment

Participants completed an online assessment that consisted of demographic information including age, sex, nationality and student status. They also indicated past year medication and illicit drug use and whether they were a current smoker. Historical alcohol consumption questions included age at which they first consumed alcohol and age alcohol was consumed regularly. Participants then completed the 3-item Alcohol Use Disorders Identification Test (AUDIT-C; Bush *et al*., 1998), to identify potential risk for alcohol use disorder. The score on the AUDIT-C ranges from 0 to 12, with a score of 5–7 indicating increased risk, 8–10 higher risk and 11–12 possible alcohol dependence.

#### Smartphone-based app

The instant messaging platform, WhatsApp was utilized for participants to report on their alcohol consumption practices. WhatsApp is freely available for both Android and iOS smartphone platforms. Its user-friendly interface allows voice, text, picture and video content to be sent to selected contacts using end-to-end encryption. WhatsApp is the world’s most popular mobile messaging application, with 2 billion users (WhatsApp, 2021) and >100 billion messages sent every day (TechCrunch, 2020). In the UK, 80% of adults aged 18–24 years old are frequent WhatsApp users (Statistia, 2021). Despite its popularity and potential to collect real-time data, no known studies have used this app within alcohol consumption research.

Participants were asked to report on their recreational alcohol consumption occasion in real-time as it happened or shortly after. This involved participants sending a message to the research team via WhatsApp stating ‘alcohol consumption started’. This would be accompanied by a photo of the alcoholic beverage consumed, along with a short description to include brand, volume, price and location. Participants continued to report their alcohol consumption in real-time as the drinking occasion progressed. The short description provided allowed the research team to accurately record the quantity of alcohol consumed using standardized UK units (Drinkaware, 2020). Participant responses were monitored by a research assistant in real-time with any non-standard alcohol drinks queried with participants for further information. Consensus was reached on any remaining unidentifiable alcoholic drinks by two researchers using the information available and comparing with available drink menus from the area. This was only required for 2.8% of all alcoholic drinks consumed.

Data from the 2-week familiarization period indicated that 68% of all recreational alcohol consumption occasions began between 7 and 9 pm. Therefore, a daily prompt was sent at 8 pm to remind participants to record their consumption if they planned on drinking alcohol or to recollect the time and amount consumed if they had forgotten to doso.

Participants were asked to notify the research team when a recreational drinking occasion had terminated by sending a message via WhatsApp stating ‘alcohol consumption finished’. This allowed the duration of the drinking occasion to be determined.

If participants reported on a recreational alcohol consumption occasion, they were sent a follow-up questionnaire the next day. Here, participants were asked to report using a dichotomous response format (yes, no) whether they had engaged in 25 risk-taking behaviours (Peacock *et al*., 2012) during the drinking occasion. The risk-taking behaviours selected represented several themes including licit and illicit drug use, sexual practices, motor vehicle behaviour, financial outcomes, aggressive behaviour, mental and physical distress, injury or harm and other antisocial behaviours. At this point participants were also provided with an opportunity to review their previous night responses to include any further drinks consumed or to reassess the time of termination of their drinking occasion.

If participants had not reported on a recreational alcohol consumption occasion, they were sent a prompt the following day at 11 am to confirm that they had not consumed alcohol. The purpose of this prompt was to assess compliance. If they had consumed alcohol, they were provided with an opportunity to report on the amount consumed and the duration of the consumption occasion, as well as reporting any risk-taking behaviours. Non-compliance with real-time alcohol consumption reporting occurred on only 1.4% of all drinking occasions.

#### Follow-up visit

Here, participants completed alcohol consumption questions adapted from the QDS (Sobell *et al*., 2003). These questions comprised past 30 days quantity of alcohol consumption on a typical drinking occasion, the number of alcohol consumption days and the number of binge drinking days (i.e. more than four (women) or five (men) alcohol units consumed). In addition, for their past 30 days heaviest drinking occasion, participants reported on the number of alcohol units consumed and the duration of the drinking session. These questions were answered separately for both AO and AMED drinking occasions. Mixing was defined as consuming an energy drink within a time period of ±2 h of alcohol consumption. Finally, participants were asked to report whether or not they had engaged in risk-taking behaviours on AO and AMED drinking occasions during the past 30 days and the frequency for each reported behaviour. All participants attended the laboratory and completed their retrospective recall of alcohol consumption (a-QDS) and risk-taking behaviours with reference to the same 30-day period within 3 days of this data collection period.

### Statistical analysis

To allow for a comparison between EMA and a-QDS data collection methods, EMA data from the same 30 days following the familiarization period and inclusive of four weekends were extracted and collated for the 52 completing participants.

Firstly, consumption occasions were categorized as AO or AMED depending on whether participants had reported consuming energy drink within a time period of ±2 h of alcohol consumption during that drinking occasion. Drinking occasions were also categorized as binge drinking or non-binge drinking depending on whether the occasion reported consuming more than four (women) or five (men) alcohol units.

Data related to the number of drinking days and binge drinking days were aggregated across AO and AMED drinking occasions for each individual. The numbers of alcohol units per usual drinking occasion were calculated separately for AO and AMED occasions by dividing the total standard drinks consumed by the number of drinking occasions. The heaviest drinking AO and AMED occasion were identified for each participant by selecting the occasions reporting the highest number of alcohol units consumed. The hours of drinking were computed from the time the participant informed the researcher of the commencement of this drinking occasion to the termination of this drinking occasion to the nearest quarter of an hour. Risk-taking behaviours were summed across all AO or AMED drinking occasions to provide a total number of risk-taking behaviours. In order to adjust for the fewer reported AMED drinking occasions compared with AO drinking occasion, the total number of risk-taking behaviours were also divided by the total number of drinking occasions.

To assess the EMA data collection method for compliance and reactivity the data was split into 6 weeks of data collection. This consisted of the 2-week familiarization period and 4 weeks of the 30-day data collection period, exclusive of the final 2 days.

Once collated, data were analysed using IBM SPSS Version 26 (IBM SPSS Statistics 2019 Armonk, NY: IBM Corp.). Demographic data were first explored using appropriate descriptive statistics. The alcohol consumption and risk-taking data were then checked for violation of parametric assumptions. Outliers were identified in the data, as assessed by inspection of boxplots for values greater than 1.5 box-lengths from the edge of the box. However, these values were considered genuine values within the expected range captured in previous studies (Johnson *et al*., 2016a, 2016b). These outliers were therefore retained for analysis. The data were also not normally distributed as assessed by Shapiro–Wilk’s test (*P* < 0.05).

Given that the assumptions for a one-way repeated measures parametric analysis of variance were not met, compliance and reactivity for the EMA data collection method was assessed using a Freidman nonparametric test. Overall significant differences were followed by pairwise Wilcoxon signed-rank tests with Bonferroni correction to control for type I errors, in order to indicate where the differences were located. Differences were regarded as significant at *P* < 0.003 (0.05/15).

In line with the a priori research questions, statistical analyses for the alcohol consumption and risk-taking data were based on comparing consumption occasion (AO versus AMED) and data collection method (EMA versus a-QDS). Therefore, the nonparametric Wilcoxon signed-rank test for paired comparisons was conducted. To control for type I errors from the number of paired comparisons made, Bonferroni adjustments were again made with differences regarded as significant at *P* < 0.0125 (0.05/4). Appropriate effect sizes (Rosenthal, 1991) are reported for all significant findings. Following Cohen (2013), effect sizes 0.1 or below were considered ‘small’, around 0.3 ‘medium’ and 0.5 or above ‘large’ in magnitude.

## RESULTS

### Sample demographics

The demographics for the completing participants are presented in Table 1. Of these 52 participants who consumed both AO and AMED during the 30-day data collection period, 57.7% were female and 42.3% were male. At the time of baseline assessment 51.9% of the sample were aged 20 years or under, 46.2% were aged between 21 and 24 years and 1.9% were aged 25 years or older. 71.2% of participants identified as white, with 11.5% Asian, 9.6% Black, 3.8% mixed ethnicity and 3.8% other. The majority of participants were studying at undergraduate level (80.8%), with the remaining 19.2% studying at postgraduate level. These demographics broadly reflected that of the student population in the UK (Higher Education Statistics Agency, 2021).

**Table 1:** Demographics of participants who consumed AO and AMED during 30-day data collection period

Characteristics	AO and AMED consumers (*N* = 52)
Gender	
Female	57.7
Male	42.3
**Age (years)**	
20 and under	51.9
21–24	46.2
25 and older	1.9
**Ethnicity**	
White	71.2
Asian	11.5
Black	9.6
Mixed	3.8
Other	3.8
**Student status**	
Undergraduate	80.8
Postgraduate	9.2
Medication use (past year)	23.1
Illicit drug use (past year)	30.8
Current smoker (% yes)	34.6
Age first consumed alcohol (years)	14.2 (1.5)
Age consumed alcohol regularly (years)	17.2 (1.1)
Total AUDIT-C Score (0–12)	6.9 (1.8)

*Note:* % or mean (SD) are shown.

*Abbreviations:* AO, alcohol-only; AMED, alcohol mixed with energy drinks; AUDIT-C, alcohol use disorder identificationtest.

Past year medication and illicit drug use was reported as 23.1 and 30.8%, respectively, and 34.6% identified as current smokers. The mean age at which participants first consumed alcohol was 14.2 years, and the mean age at which they began consuming alcohol regularly was 17.2 years. The mean AUDIT-C score was 6.9, indicating increasing risk for alcohol-related harm. These findings are similar to those reported in previous AMED research using the same student population (Johnson *et al*., 2016a, 2016b).

### Smartphone-based app compliance and reactivity

**Figure 2:**
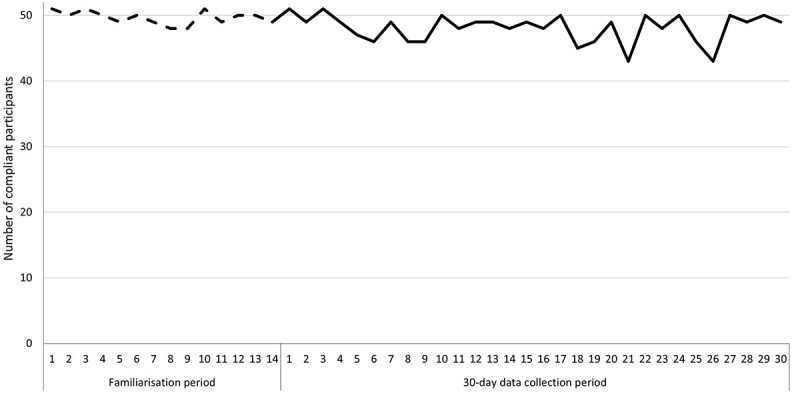
Daily number of participants compliant with study protocol.

Participants were recorded as compliant with the study protocol if they had either provided information about their alcohol consumption (event-contingent) or responded to the next day prompt to confirm that they had not consumed alcohol the previous day (notification-contingent). On average, participants were compliant on 13.4 days out of 14 during the familiarization period (95.5%) and on 27.8 days out of 30 during the data analysis period (92.6%). Figure 2 indicates the number of compliant participants each day. A Freidman test showed that the number of days participants were compliant was statistically significantly different between data collection weeks [*X*^2^(5) = 20.205, *P* = 0.001]. Post hoc analysis with a Bonferroni adjustment revealed that the number of days participants were compliant were significantly higher during Week 1 (*Mdn* = 7.0) of the familiarization period compared with Week 3 (*Mdn* = 6.0, *P* = 0.001) and Week 4 (*Mdn* = 7.0, *P* = 0.002) of the data analysis period. No further differences in the number of days participants were compliant were found between data collection weeks.

In total, 1728.6 alcohol units were captured via the app during the 2-week familiarization period, and 3358.6 alcohol units across the 30-day data analysis period. A Freidman test showed that the total number of alcohol units consumed by participants did not significantly differ between data collection weeks [*X*^2^(5) = 1.320, *P* = 0.933]. In addition, there were no significant differences in the total number of days consuming alcohol between the data collection weeks [*X*^2^(5) = 1.750, *P* = 0.883].

### Comparison of AO and AMED drinking occasions

To establish whether mixing energy drinks with alcohol had an impact on total alcohol consumption, within-subject comparisons were performed for AMED drinking occasions versus AO drinking occasions, across both EMA and a-QDS data collection methods.

As shown in Table 2 the analysis revealed that when collected via EMA, participants reported significantly more overall drinking days (*P* < 0.001) and binge drinking days (*P* < 0.001) on AO drinking occasions compared with AMED drinking occasions in the past 30 days. Although there were no significant differences in the number of alcohol units consumed during an average AO versus AMED drinking occasion (*P* = 0.128), significantly more alcohol was consumed on the past 30 days heaviest AO drinking occasion compared with the past 30 days heaviest AMED drinking occasion (*P* < 0.001). No significant differences were found in the duration of the heaviest AO and AMED drinking occasions (*P* = 0.606). The total number of risk-taking behaviours experienced on AO occasions was significantly higher than those experienced on AMED occasions (*P* < 0.001). However, after taking into account the number of drinking occasions this was no longer statistically significant (*P* = 0.015).

**Table 2:** Comparison of alcohol consumption and risk-taking behaviours on AO and AMED occasions collected via EMA and a-QDS

	EMA					a-QDS				
	AO	AMED	*z*	*P*	*r*	AO	AMED	*z*	*P*	*r*
Past 30 days usual drinking occasions										
Number of alcohol units	10.0	10.0	−1.52	0.128	–	8.0	7.5	−1.04	0.301	–
Number of drinking days (alcohol)	4.0	2.0	−6.12	< 0.001	0.60	4.0	1.0	−6.12	< 0.001	0.60
Binge drinking days (>4/5 alcoholic drinks)	4.0	1.0	−5.87	< 0.001	0.58	4.0	1.0	−5.86	< 0.001	0.57
Past 30 days heaviest drinking occasion										
Number of alcohol units	15.0	10.5	−4.80	< 0.001	0.47	12.5	8.0	−4.90	< 0.001	1.08
Hours of drinking	4.25	4.5	−0.52	0.606	–	4.0	4.0	−0.72	0.472	–
Risk-taking behaviours past 30 days										
Total number of risk-taking behaviours	6.0	1.0	−5.80	< 0.001	0.57	4.5	1.0	−5.67	< 0.001	0.56
Total number of risk-taking behaviours/number of drinking occasions	1.4	1.0	−2.43	0.015	–	1.2	1.0	−2.78	0.005	0.27

*Notes:* Median values shown. Effect sizes provided for significant differences following Bonferroni adjustment for the number of paired comparisons (*P* < 0.0125). One alcohol unit = 8 g/10 ml of pure alcohol.

*Abbreviations:* AO, alcohol-only; AMED, alcohol mixed with energy drinks; EMA, ecological momentary assessment; a-QDS, adapted-Quick Drinking Screen.

Similar patterns of significant differences were also found for the a-QDS, indicating a higher frequency in the number of overall drinking days (*P* < 0.001), binge drinking days (*P* < 0.001) and higher alcohol consumption during the heaviest consumption occasion (*P* < 0.001) on AO occasions compared with AMED occasions. The total number of risk-taking behaviours were also significantly higher on AO occasions compared with AMED occasions (*P* < 0.001), and this remained statistically significant after taking into account the number of drinking occasions (*P* = 0.005). Effect sizes were mainly medium to large in magnitude (ranging between 0.27 and 1.08) for statistically significant comparisons.

### Comparison of EMA and a-QDS data collection methods

To establish whether there was a difference in the frequency and quantity of alcohol reported via EMA or a-QDS, within-subjects comparisons were performed separately for AO and AMED drinking occasions.

Table 3 demonstrates that for AO drinking occasions, participants reported consuming significantly more alcohol units on an average (*P* < 0.001) and heaviest drinking occasion (*P* < 0.001) via EMA compared with a-QDS. However, there was no difference in the reported duration of the heaviest drinking occasion (*P* = 0.230). There were also no significant differences in the overall number of AO drinking days (*P* = 0.074) or AO binge drinking days (*P* = 0.015) when reported via EMA compared with a-QDS. The total number of risk-taking behaviours reported for AO occasions was significantly higher when reported via EMA compared with a-QDS (*P* < 0.001) and this remained significant when taking into account the number of drinking occasions reported (*P* < 0.001).

**Table 3:** Comparison of alcohol consumption and risk-taking behaviours collected via EMA and a-QDS for AO and AMED occasions

	AO					AMED				
	EMA	a-QDS	*z*	*P*	*r*	EMA	a-QDS	*z*	*P*	*r*
**Past 30 days usual drinking occasions**										
Number of alcohol units	10.0	8.0	−4.66	< 0.001	0.46	10.0	7.5	−4.75	< 0.001	0.47
Number of drinking days (alcohol)	4.0	4.0	−1.79	0.074	–	2.0	1.0	−1.41	0.157	–
Binge drinking days (>4/5 alcoholic drinks)	4.0	4.0	−2.43	0.015	–	1.0	1.0	−2.31	0.021	–
**Past 30 days heaviest drinking occasion**										
Number of alcohol units	15.0	12.5	−4.57	< 0.001	0.45	10.5	8.0	−4.57	< 0.001	0.45
Hours of drinking	4.25	4.0	−1.20	0.230	–	4.5	4.0	−1.71	0.088	–
**Risk-taking behaviours past 30 days**										
Total number of risk-taking behaviours	6.0	4.5	−4.70	< 0.001	0.46	1.0	1.0	−2.33	0.020	–
Total number of risk-taking behaviours/number of drinking occasions	1.4	1.2	−3.70	< 0.001	0.36	1.0	1.0	−2.04	0.041	–

*Notes:* Median values shown. Effect sizes provided for significant differences following Bonferroni adjustment for the number of paired comparisons (*P* < 0.0125). One alcohol unit = 8 g/10 ml of pure alcohol.

*Abbreviations:* AO, alcohol-only; AMED, alcohol mixed with energy drinks; EMA, ecological momentary assessment; a-QDS, adapted-Quick Drinking Screen.

A similar pattern of significant differences was also found for AMED drinking occasions. Participants reported consuming a higher number of alcoholic drinks on an average (*P* < 0.001) and heaviest (*P* < 0.001) drinking occasion. Furthermore, there were no significant differences in the overall number of AMED drinking days (*P* = 0.157) or AMED binge drinking days (*P* = 0.021) when reported via EMA compared with a-QDS. However, in contrast to AO drinking occasions, there were no significant differences in the number of risk-taking behaviours (*P* = 0.020) reported via EMA compared with a-QDS, including when adjusted for drinking occasions (*P* = 0.041). Effect sizes were mainly medium to large in magnitude (ranging between 0.36 and 0.47) for statistically significant comparisons.

In order to investigate whether this disparity in recall (more alcohol and risk-taking behaviours reported via EMA compared with a-QDS) is differentially affected by the type of consumption occasion (AO versus AMED) a further analysis was performed. This compared the difference in the reported alcohol consumption and risk-taking behaviours between the two data collection methods across the consumption occasions. Table 4 shows that there were no significant differences in the accuracy in recall (EMA versus a-QDS) across consumption occasions (AO versus AMED) for all alcohol consumption questions (*P* > 0.05). Thus, the increased amount of alcohol reported via EMA compared with a-QDS was consistent across drinking occasions (AO and AMED).

**Table 4:** Comparison of accuracy in recall (EMA versus a-QDS) of past 30 days alcohol consumption and risk-taking behaviours for AO and AMED occasions

	AO (EMA—a-QDS)	AMED (EMA—a-QDS)	*z*	*P*	*r*
**Past 30 days usual drinking occasions**					
Number of alcohol units	1.89	2.0	−0.54	0.592	–
Number of drinking days (alcohol)	0.00	0.00	−0.39	0.695	–
Binge drinking days (>4/5 alcoholic drinks)	0.00	0.00	−1.53	0.127	–
**Past 30 days heaviest drinking occasion**					
Number of alcohol units	2.0	1.5	−0.26	0.799	–
Hours drinking	0.25	0.25	−0.31	0.753	–
**Risk-taking behaviours past 30 days**					
Total number of risk-taking behaviours	1.0	0.00	−3.70	< 0.001	0.36
Total number of risk-taking behaviours/number of drinking occasions	0.16	0.00	−1.09	0.274	–

*Notes:* median values shown. One alcohol unit = 8 g/10 ml of pure alcohol.

*Abbreviations:* AO, alcohol-only; AMED, alcohol mixed with energy drinks; EMA, ecological momentary assessment; a-QDS, adapted-Quick Drinking Screen.

There was an increase in the difference between EMA and a-QDS for the total number of risk-taking behaviours, with significantly more reported on AO occasions compared with AMED occasions (*P* < 0.001) reflecting the relatively greater number reported under AO with EMA. This had a medium effect size (0.36). However, this difference was no longer significant when the number of drinking occasions was taken into account (*P* = 0.274).

## DISCUSSION

The aim of the current study was to investigate alcohol consumption and risk-taking behaviours on AO and AMED occasions using both prospective (EMA) and retrospective (a-QDS) methods. It was reliably found across both data collection methods that alcohol is consumed more frequently and at higher quantities on the heaviest drinking occasions when consumed alone compared with when it is mixed with energy drinks. In addition, significantly more overall risk-taking behaviours were experienced on AO occasions compared with AMED occasions. These findings are comparable with previous within-subject survey research that have also utilized the a-QDS or other retrospective methods (Verster *et al*., 2018). The significance of this study is that it is the first to utilize EMA to capture AMED consumption in real-time. The consistency in findings across both data collection methods further strengthens the conclusion that mixing alcohol with energy drinks seems unlikely to increase alcohol consumption or associated risk-taking behaviour by itself, and that as demonstrated by between-subject research (O’Brien *et al*., 2008; Woolsey *et al*., 2010; Brache and Stockwell, 2011; de Haan *et al*., 2012; Eckschmidt *et al*., 2013; Lubman *et al*., 2013; Trapp *et al*., 2013; Vida and Racz, 2015; Woolsey *et al*., 2015a, 2015b; Johnson *et al*., 2016a), AMED consumption seems to be just one manifestation of an underlying trait for greater alcohol consumption along with a cluster of other risky behaviours (Verster *et al*., 2012).

A further aim of the study was to compare prospective (EMA) and retrospective (a-QDS) data collection methods to examine the claim that recall bias and other factors could differentially affect memory of AMED occasions over AO occasions (Linden-Carmichael *et al*., 2018). It was found that the number of alcohol units consumed on the average and heaviest drinking occasions, and overall risk-taking behaviours were significantly higher when reported via a prospective method (EMA) compared with a retrospective method (a-QDS). However, there were no significant differences in the frequency of usual and heaviest drinking occasions, nor hours of drinking for heaviest drinking occasions reported via EMA and a-QDS. These findings were consistent across both AO and AMED drinking occasions. This demonstrates that retrospective recall of alcohol consumption quantity is susceptible to recall bias in the form of under-reporting but that this bias is not differentially affected across consumption occasions. This finding is important as it provides some confidence in the direction of findings from previous studies using retrospective methods to assess AMED and AO consumption, with the caveat that reported quantity may be less for both AMED and AO when reported retrospectively than was actually consumed. Furthermore, there was reduced recall of risk-taking behaviours with AO when unadjusted for drinking occasions. This underreporting is consistent with previous research that has compared retrospective and prospective data collection methods (Heeb and Gmel, 2005; Monk *et al*., 2015; Dulin *et al*., 2017; Poulton *et al*., 2018). As well as recall bias, these differences have been explained as being due to participants’ difficulty in conceptualizing their usual consumption occasion, especially if their intake is highly variable across drinking occasions, with a tendency to overlook occasional high drinking occasions (Del Boca and Darkes, 2003; Stockwell *et al*., 2004). Consequently, participants tend to report modal rather than average consumption in response to retrospective surveys (Utpala-Kumar and Deane, 2010).

There are a number of limitations to the current study that need to be acknowledged. Firstly, whilst EMA studies address some of the limitations of retrospective surveys they do have their own drawbacks. EMA relies on participants accurately and consistently reporting their alcohol consumption whilst under the influence. Therefore, it is plausible that engagement with the app decreases as inebriation increases. In addition, societal stigma of high alcohol consumption among students in the UK (NUS Alcohol Impact, 2020) may have led to over-reporting with participants inflating their consumption to match this stereotype. Conversely, as shown in other studies (McCambridge and Kypri, 2011; Smith *et al*., 2017) participants may have reduced their alcohol consumption, simply as a consequence of knowing they were being measured. Further, engagement with the app may have drawn their attention to their current consumption and this may have influenced overall consumption. However, this may be predicted to moderate rather than exacerbate consumption levels.

Although overall compliance with the smartphone-based app was high, with a 92.6% response rate, it is possible that as the data collection period progressed participants may have found continual use of the app burdensome reducing engagement. This could have then resulted in participants recording a ‘No’ response even on occasions when alcohol was consumed. Further analysis of the current dataset will be conducted to examine how the frequency and quantity of alcohol consumed changed over time using a multilevel modelling approach. In addition, engagement may have reduced with higher levels of consumption and increased inebriation, so that the highest consumption levels may have been under-represented or recorded consumption reduced in comparison to actual consumption. However, this can only be resolved by third party methods of real time consumption recording in order to triangulate and confirm the accuracy and reliability of first party methods.

With regard to the smartphone-based app used, participants provided pictures and a short description of the alcoholic beverages consumed. The advantage of this is that participants were not limited to pre-defined options. For the vast majority of alcoholic beverages reported it was easy to identify the associated standardized UK alcohol content, however on some occasions this required some interpretation and consensus amongst researchers. This was particularly the case in non-licensed locations, usually during pre-drinking or after-parties, when non-standard serve sizes were consumed. Inaccuracies relating to non-standard serving sizes would, however, likely to be reported across both AMED and AO drinking occasions, as well as prospective and retrospective methods.

To overcome the above limitations, future EMA research could utilize wearable transdermal biosensor devices that allow discrete, continuous, objective monitoring of alcohol consumption in real-time. Although these devices are currently in use within the criminal justice system to monitor alcohol abstinence, further development is required to validate the accuracy of continuous monitoring across the BAC curve (Fairbairn and Kang, 2021).

With regard to the sample obtained, whilst attempts were made to recruit participants who were frequent AMED consumers a significant percentage (45.3%) did not report an AMED drinking occasion during the 30-day data analysis period. This may have been influenced by the timing of data collection, with the inclusion criteria of two AMED occasions within the past 2 months falling over the festive period (December), and the data collection in the new year period that followed when it is popular to reduce consumption. Indeed, one of the most frequently reported motives for AMED consumption is ‘to celebrate a special occasion’ (Johnson *et al*., 2016a). Future research could overcome this limitation by including a more nuanced definition of AMED consumer and conducting the study in bursts throughout the year to capture seasonal differences. However, a further limitation of this approach is the significant resource requirements associated with repeated data collection blocks for 30 day or longer periods.

Lastly, the sample consisted of university students from one geographical area of the UK, therefore it is not possible to generalize these results beyond this population. Additional studies are required, both in different areas of the UK and in other countries, before any definitive conclusions can bemade.

## CONCLUSION

In summary, this is the first investigation that we are aware of to successfully utilize EMA to provide real time data for both alcohol alone and AMED consumption in the same participants. This has revealed the greater sensitivity of the EMA technique in comparison to recall methods that are impacted by memory bias or errors, and are particularly susceptible to alcohol induced impairment. The current study demonstrates that mixing alcohol with energy drinks does not increase overall alcohol consumption and risk-taking behaviours compared with consuming alcohol alone. In addition, although retrospective (a-QDS) recall of alcohol consumption quantity is susceptible to under-reporting compared with prospective methods (EMA), this is not differentially affected across consumption occasions. Overall, the findings of this study suggest that public health policy might take into account retrospective under-reporting, and should focus on addressing excessive alcohol consumption per se. Future research should utilize EMA for a more fine-grained and accurate investigation into the way in which drinking occasions unfold to examine the socio-environmental factors that contribute to heavy and risky alcohol consumption.

## AUTHORS’ CONTRIBUTIONS

S.J.J., J.C.V., C.A. conceptualized and designed the study; S.J.J. was involved in data collection and data analysis, and led the drafting of the original manuscript; S.J.J., J.C.V., C.A. took the responsibility of writing—review and editing for its final content; J.C.V., C.A. did supervision and C.A. was involved in funding acquisition. All authors have read and agreed to the published version of the manuscript.

## Data Availability

The data are available from the corresponding author upon reasonable request.
