# Corrigendum to: A Comparison Between Ecological Momentary Assessment and the Adapted-Quick Drinking Screen: Alcohol Mixed With Energy Drinks

**DOI:** 10.1093/alcalc/agac007

**Published:** 2022-02-15

**Authors:** Sean J Johnson, Joris C Verster, Chris Alford

**Affiliations:** Centre for Trials Research, Cardiff University, Cardiff CF14 4YS, UK; Psychological Sciences Research Group, University of the West of England, Bristol BS16 1QY, UK; Division of Pharmacology, Utrecht Institute for Pharmaceutical Sciences (UIPS), Utrecht University, Utrecht 3584CG, The Netherlands; Centre for Human Psychopharmacology, Swinburne University, Melbourne, VIC 3122, Australia; Psychological Sciences Research Group, University of the West of England, Bristol BS16 1QY, UK; Centre for Human Psychopharmacology, Swinburne University, Melbourne, VIC 3122, Australia

In the originally published version of this manuscript, there was an error in the y axis label for Figure 2 which should read: “Number of compliant participants” instead of “Number of compliance participants”. Notes to Table 2 should read: “*Abbreviations*: AO, alcohol-only; AMED, alcohol mixed with energy drinks; EMA, ecological momentary assessment; a-QDS, adapted-Quick Drinking Screen.” instead of: “*Abbreviations*: AO, alcohol-only; AMED, alcohol mixed with energy drinks; EMA, ecological momentary assessment; a-QDS, adapted-Quick Drinking Screen, *Mdn*, median.” Notes to Table 4 should read: “*Abbreviations*: AO, alcohol-only; AMED, alcohol mixed with energy drinks; EMA, ecological momentary assessment; a-QDS, adapted-Quick Drinking Screen.” instead of “*Abbreviations*: AO, alcohol-only; AMED, alcohol mixed with energy drinks; EMA, ecological momentary assessment; a-QDS, adapted-Quick Drinking Screen; M, mean; SD, standard deviation.”. These errors have now been corrected within the article online.

